# Delayed Absorption Superabsorbent Polymer for Strength Development in Concrete

**DOI:** 10.3390/ma15082727

**Published:** 2022-04-07

**Authors:** Yuka Morinaga, Yuya Akao, Daisuke Fukuda, Yogarajah Elakneswaran

**Affiliations:** 1 Division of Sustainable Resources Engineering, Faculty of Engineering, Hokkaido University, Sapporo 060-8628, Japan; morinaga@eng.hokudai.ac.jp (Y.M.); d-fukuda@frontier.hokudai.ac.jp (D.F.); 2 Industrial & Household Chemicals Research Department, Industrial & Household Solutions Division, Nippon Shokubai, Osaka 564-0034, Japan; yuya_akao@shokubai.co.jp

**Keywords:** superabsorbent polymer, compressive strength, pore structure, hydration rate, concrete

## Abstract

Superabsorbent polymers (SAPs) are used as internal curing agents in cementitious materials, which reduce autogenous shrinkage in concrete as they have a low water-to-cement ratios and improve the freeze–thaw resistance. However, the compressive strength of concrete may also be reduced due to additional voids in the hydrated cement matrix. In this study, we fabricated a delayed absorption type of SAP (I-SAP) composed of cross-linked modified acrylate and studied its absorption characteristics and effect on compressive strength after 28 days. Furthermore, the effect of curing conditions on the strength of concrete and hydrated cement paste with SAP were investigated. The absorption capacity of I-SAP in the synthetic pore solution and deionised water was examined and compared with that of a conventional SAP, and the former was absorbed more by I-SAP. The results revealed that the compressive strength of concrete increased with the addition of I-SAP, particularly with the curing condition of 60% RH. Although the compressive strength of hydrated cement paste with I-SAP reduced in water or sealed curing conditions, no loss of strength in the paste cured at 60% RH was seen. The cement matrix densification due to hydration of belite around the SAP surface is the main mechanism for strength development in concrete cured at sealed and 60% RH. However, the voids formed by SAP control the compressive strength of hydrated paste.

## 1. Introduction

Superabsorbent polymers (SAPs) are hydrophilic networks capable of absorbing and storing large amounts of water or aqueous solutions and are used in various applications. Recently, SAPs have been used as internal curing agents in cementitious materials for mitigating autogenous shrinkage in ultra-high-performance concrete (UHPC) and HPC, as they have a low water-to-cement ratio [1,2,3]. SAP not only supplies water from the inside for the hydration reaction of cement in a low water–cement ratio, but also it provides water when there is not enough for the hydration reaction of cement due to its evaporation during concrete curing in an environment with low relative humidity [4]. The addition of SAP is reported to effectively improve the autogenous shrinkage, enhance freezing and thawing resistances, reduce drying shrinkage, increase self-healing, reduce the formation of micro-cracking, and enhance the durability of cementitious materials [5,6,7,8,9,10,11,12,13,14,15,16]. In addition to these positive effects, the inclusion of SAP can significantly change the rheology. Particularly, when SAP is added to mortar, the slump flow is decreased, owing to the reduction of the water available for mixing. Conventional SAP absorbs water in a few minutes [17]; thus, additional water or superplasticizer needs to be added to improve the slump flow [11,12,18,19], which influences the mechanical properties.

Diverse results have been reported in the literature regarding the strength of cementitious materials with SAP. Many studies have reported that the compressive strength of cementitious materials remains constant or is reduced with the addition of SAP [3,10,11,20,21,22,23,24,25,26,27,28,29]. The addition of extra water to the SAP-added concrete to compensate for workability increases the pore water and reduces the concrete strength. Moreover, SAP creates additional voids in the cement matrix, thus reducing its strength. This effect could be partially offset by the additional hydration due to internal curing. On the other hand, strength increments with SAP addition have been reported by others. Bentz et al. [30] reported that SAP-added mortar showed a higher strength after 28 days, and AzriJafari et al. [31] showed an increase in the strength of silica fume added to concrete. In addition, Gao et al. [32] demonstrated the strength development of aluminous salt cement paste with SAP. The positive or negative effect of SAP on the compressive strength depends on the SAP type and its added amount, water-to-binder ratio, curing conditions, testing age, type of sample (concrete, mortar, or paste), and material composition [33]. Two main reasons for the decrease in the strength of concrete can be attributed to the formation of void space after SAP releases water and the increase in the water-to-cement ratio in the hardened cement paste, due to the addition of excess water. These are the major issues in the conventional SAPs, which were not examined properly in previous studies.

Several solutions have been proposed to limit the water absorption of mixing water and to reduce the loss of compressive strength [33]. When the water absorption rate of SAP is very low, it shows a smaller effect on rheology. Based on previous studies regarding the negative effects of SAP, further studies on the development of SAP and its influence on compressive strength are necessary. If SAP-added concrete could potentially be produced without adding extra water, the autogenous shrinkage can be mitigated, and drying shrinkage can be reduced while maintaining its mechanical properties. SAP supplies water from the inside, so it is also expected to increase the strength of concrete in a dry environment. Therefore, we examined the effect of a newly developed delayed-water-absorption SAP on the absorption characteristics and compressive strength of concrete and cement paste. The absorption behaviour of the new SAP was compared with that of a conventional SAP. The influence of curing conditions on the compressive strength of SAP-added concrete and cement paste was examined and compared with that of the control specimen. Finally, the mechanism of concrete strength development and its reduction in cement paste were discussed with respect to hydration, porosity, and microstructure.

## 2. Materials and Methods

### 2.1. Materials and Sample Preparation

Here, ordinary Portland cement (OPC, Taiheiyo Cement Corporation, Tokyo, Japan), chemical composition given in Table 1, was used to prepare the cement paste and concrete. A delayed absorption type SAP was developed and used in this study (hereafter called I-SAP), which was composed of cross-linked modified acrylate (Nippon Shokubai, Osaka, Japan). It had a mean particle size (d50) of 400 μm and mainly consisted of the monomer modified-acrylates and cross-linker. A conventional SAP (C-SAP) composed of cross-linked acrylate was used for comparison. The SAP addition to the cement was 0.3% by weight of cement. Cement paste was prepared with a water-to-cement ratio of 0.45 and a flow of 160 ± 10 mm, which were achieved by adding a polycarboxylic acid ether-type superplasticizer (SP) (Mastergrenium SP 8SV, BASF, Tokyo, Japan). The cement was mixed with water in a mortar mixer for 60 s at a low speed and then for 120 s at a high speed. The prepared cement slurry was stored in a plastic bottle and agitated with a spatula for few hours at intervals of 1 h to prevent bleeding. Afterwards, this slurry was poured into nine moulds (diameter 50 mm × 100 mm height), sealed with a plastic sheet, and then stored for 24 h at 20 °C. The samples were demoulded, three of which were cured in water, another three samples were placed under sealed conditions, and the remaining three in a 60% RH environment. All specimens were cured at 20 °C for 28 days and then subjected to various measurements. It is worth to mentioned that an average of three specimen results is presented in the figures. Moreover, this study focused on the changes of later stage compressive strength and therefore, a curing time of 28 days was selected for the cement paste and concrete.

The mix proportions of concrete and its fresh properties are listed in Table 2. The initial materials were selected according to JIS A 5308, and concrete formulations were determined by referring to JIS A 6204. Concrete fabricated with SAP is denoted as SAP–concrete, and that without SAP is the reference. The concrete was cast with a dual-axle revolving-paddle mixer with a capacity of 50 L. Initially, cement, fine aggregate, coarse aggregate, and SAP were mixed for 10 s without water, then water with SP and defoamer (ADEKA, Tokyo, Japan) was added and mixed for 90 s. The dosage of SP was adjusted to achieve a target slump value of 22 ± 1 cm. The air content was controlled in the range of 4.5 ± 0.5 vol.% using both SP and defoamer. The amounts of SP and defoamer were subtracted from that of mixing water. Both coarse and fine aggregates were used under saturated surface-dry conditions.

### 2.2. Experimental Procedure

#### 2.2.1. Particle Distribution of SAP

The particle size distribution of the SAP was determined using laser diffraction (Malvern PANalytical Mastersizer 3000, Malvern, UK). The water absorption capacity of SAPs in synthetic pore solution and deionised water was determined using the tea-bag method [11,21,34,35]. The synthetic pore solution was prepared using CaSO_4_·2H_2_O, Na_2_SO_4_, K_2_SO_4_, and KOH, (FUJIFILM Wako Pure Chemical Corporation, Osaka, Japan) assuming that the concentrations of Ca^2+^, Na^+^, K^+^, and SO_4_^2−^ were 10, 100, 180, and 86 mM, respectively, and pH was 12.8 [36]. The tea bags were pre-wetted with the pore solution or deionised water and their masses were measured. Dried SAP gel (0.2 g), was inserted into the tea bags and immersed in the solution, which were weighed after 5, 15, 30, 60, 120, and 1440 min (additionally 240, 360, and 420 min for I-SAP in the synthetic pore solution). The absorption rate was calculated as the mass ratio of wet to dry samples.

#### 2.2.2. Fluidity of Concrete

The V-funnel (Marui, Tokyo, Japan) test was used to determine the appropriate range of filling ability, viscosity, and resistance against the segregation of concrete. Initially, the funnel was filled with approximately 12 L of concrete. After 10 ± 2 s, the gate at the bottom of the V-funnel was opened, and the time taken for the concrete to flow from the funnel to the container below was measured.

#### 2.2.3. Compressive Strength, MIP, and Air-Void in Concrete

The compressive strength of the hardened cement paste and concrete cured for 28 days was measured according to JIS A 1108, formwork JIS A 1132. A mercury intrusion porosimetry (MIP) instrument was used to determine pore size distribution. The hydrated samples were cut into small pieces (approximately 5 mm in size) and dried under vacuum. The selected parameters for mercury intrusion were a surface tension of 485 erg/cm^2^, a contact angle of 130°, and applied pressure up to 4.45 psi for measuring pores larger than 3 nm in diameter. For air void evaluation, a linear transverse method in accordance with ASTM C457/C457M was adopted for the hardened specimens from each mixture.

#### 2.2.4. μCT

The cylindrical cement paste specimens cured for 28 days were cut to a diameter of 50 mm × 40 mm height for observation under micro-focus X-ray computed tomography (µCT). The pore structures of the specimens were scanned using the cone beam mode in a µCT scanner “TOSCANER 31300µhd” (Toshiba IT & Control Systems Co., Ltd., Tokyo, Japan) with parameters (tube voltage 130 kV and tube current 124 μA). The number of projection directions used in all scans was 1500. In each projection direction, 20 consecutive scans were conducted, and the averaged projection data were used for image reconstruction to reduce the statistical noise caused by the X-ray image intensifier [37]. The number and size of voxels in each µCT image was 1024 × 1024 and 51 µm × 51 µm × 77 µm, respectively. Please refer to this study [37] for further details of the applied µCT scanner.

#### 2.2.5. XRD-Rietveld

The hydrated samples were broken into pieces using a hammer and immersed in acetone for 24 h to stop the hydration reaction [38,39]. After the acetone was completely removed by suction filtration, the samples were stored at 40 °C until they showed a stable weight. The unhydrated cement was used to calculate the hydration rate. The fine powders of the samples were blended with a 10 wt.% of corundum (α-Al_2_O_3_), which is an internal standard substance. They were analysed using X-ray diffraction (XRD), which was performed using a Rigaku MultiFlex X-ray generator (Tokyo, Japan) with Cu Kα radiation under the following conditions: tube voltage of 40 kV, tube current of 40 mA, scan speed of 1º2θ min^−1^, scan range of 2θ = 5–70°, step width of 0.02°, divergence slit of 0.5°, scattering slit of 0.5°, and receiving slit of 0.3 mm. Siroquant Version 4.0 (Sietronics, Canberra, Australia) was used for quantitative Rietveld analysis where monotrinic and triclinic C_3_S, α-C_2_S, cubic C_3_A, C_4_AF, gypsum, portlandite, ettringite, monosulfoaluminate, and corundum were assigned as targets. The number of minerals and the degree of hydration were calculated according to this study [40].

#### 2.2.6. BSE

Backscattered electron images were used to observe the hydration reaction on the surface of the SAP in the samples cured for 28 d at 60% RH. The hydrated cement paste was coarsely crushed and dried with acetone to stop the hydration reaction and then vacuum dried. After one day of vacuum drying, the specimen was cast with epoxy resin and polished with abrasive paper of grid sizes 80, 240, 480, 1000, and 1500. Afterwards, the specimens were polished with diamond paste to a roughness of 0.25 μm. Finally, they were surface-dried and sputtered with platinum. A scanning electron microscope JSM-IT200 (JEOL, Tokyo, Japan) was used at an acceleration voltage of 15 kV in the backscattered electron image mode for the measurement. The obtained BSE image was separated into the unhydrated cement part and others using brightness [41], then it was binarized. The distance from the SAP and the area fraction of the unhydrated cement were also calculated.

## 3. Results and Discussion

### 3.1. Characteristics of SAP

**Figure 1:**
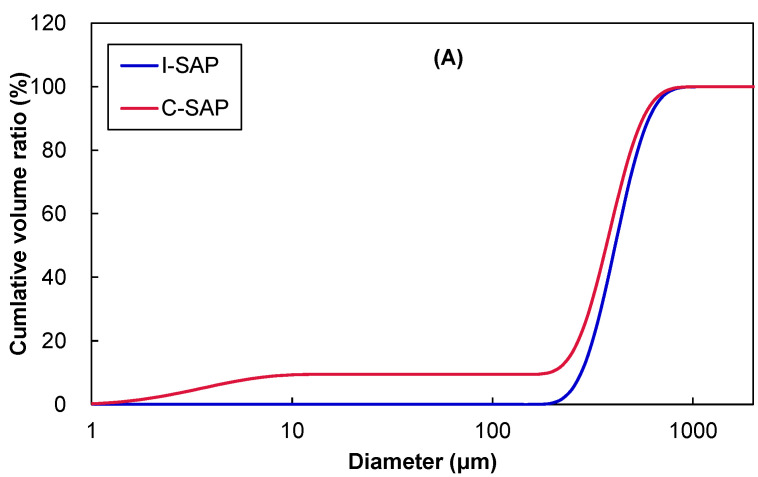

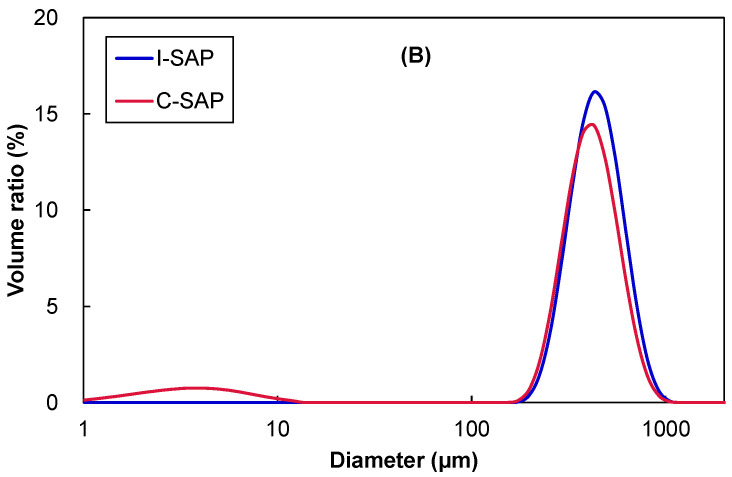
(**A**) cumulative volume, and (**B**) particle size distribution of SAPs.

The cumulative volume and particle size distribution of the SAP particles used in the water absorption experiments are shown in Figure 1. Although approximately 10% of C-SAP were 10–100 μm in size, the main peak for both SAPs was approximately 400 μm. This indicates that both SAPs have particles ranging from 200 μm–1 mm. The water absorption rate of SAP was reported to depend on the particle size, which have an inverse relationship [42]. However, the particle size distributions of both SAP particles used in this study were nearly equal.

**Figure 2:**
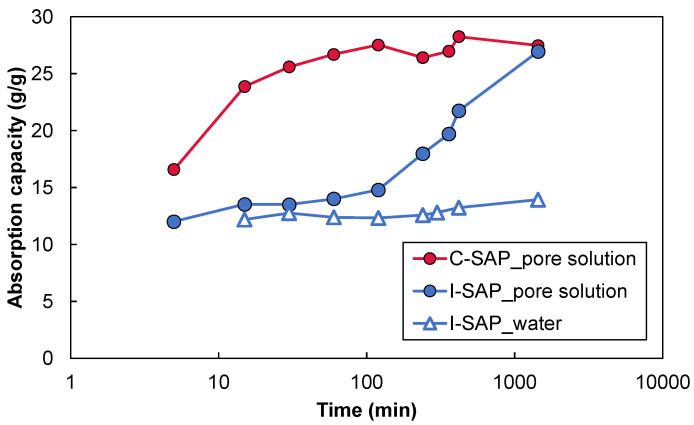
Absorption behaviour of SAPs in water and pore solution.

The water absorption behaviour of the SAP particles in the synthesised pore solution and deionised water is shown in Figure 2. Both C-SAP and I-SAP rapidly absorbed the pore solution for up to 5 min. Afterwards, the absorption ratio in C-SAP gradually increased and then remained stable after 100 min (consistent with the experimental results reported [42]). However, water absorption and swelling were suppressed from 5 min to approximately 100 min in I-SAP and then significantly increased. After 1 d, I-SAP absorbed and increased the absorption at the same level as that of C-SAP. The water absorption rate was influenced by the particle size and surrounding solution [11,21,42]. The water absorption behaviour of I-SAP in deionised water was different from that of the pore solution: I-SAP absorbed deionised water for 5 min, and afterwards it did not absorb for 24 h. The total deionised water absorption of I-SAP at 24 h was approximately half that of the pore solution. This result was contrary to the general nature of the water absorption of SAP, where SAP absorbs more water in deionised water than in the pore solution [43]. In general, the water uptake mechanism of SAPs is based on the dissociation of metal ions as water molecules enter the SAP, which ionises and negatively charges the hydrophilic groups, resulting in the formation of a concentration chain between the solution in SAP and external solution. It has been reported that the continuous flow of external solution into the SAP eventually results in a hydrogel [33,44]. The water absorption rate of SAP increases 200 to 1500 times in deionised water [21], but it decreases in high ionic strength and high pH solutions [45,46,47]. In a pore solution, the absorption rate is often under 60 [11,21]. The water absorption mechanism of I-SAP in the pore solution and deionised water cannot be explained by the mechanism reported in previous studies. More comprehensive experimental work is necessary to explain why I-SAP absorbs more in pore solutions than deionised water.

**Figure 3:**
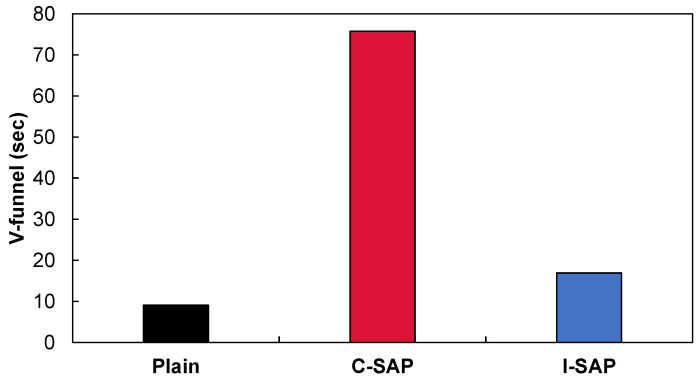
V-funnel results for concrete with and without SAP.

The V-funnel flow times of concrete with and without SAP are shown in Figure 3. The concrete with C-SAP was 8.3 times slower than that of the control mix due to the swelling of the C-SAP after contact with water. However, I-SAP showed a relatively comparable V-funnel flow as it did not absorb more water until approximately 3 h. The water absorption capacity is no more than twice the difference between SAPs, but the V-funnel flow time shows a larger difference. In other words, the addition of a general water absorbent agent has an effect that cannot be explained by only the water absorption rate; for example, the composition and surface condition of SAP could influence the viscosity.

### 3.2. Effect of Curing Method and SAP on Compressive Strength

**Figure 4:**
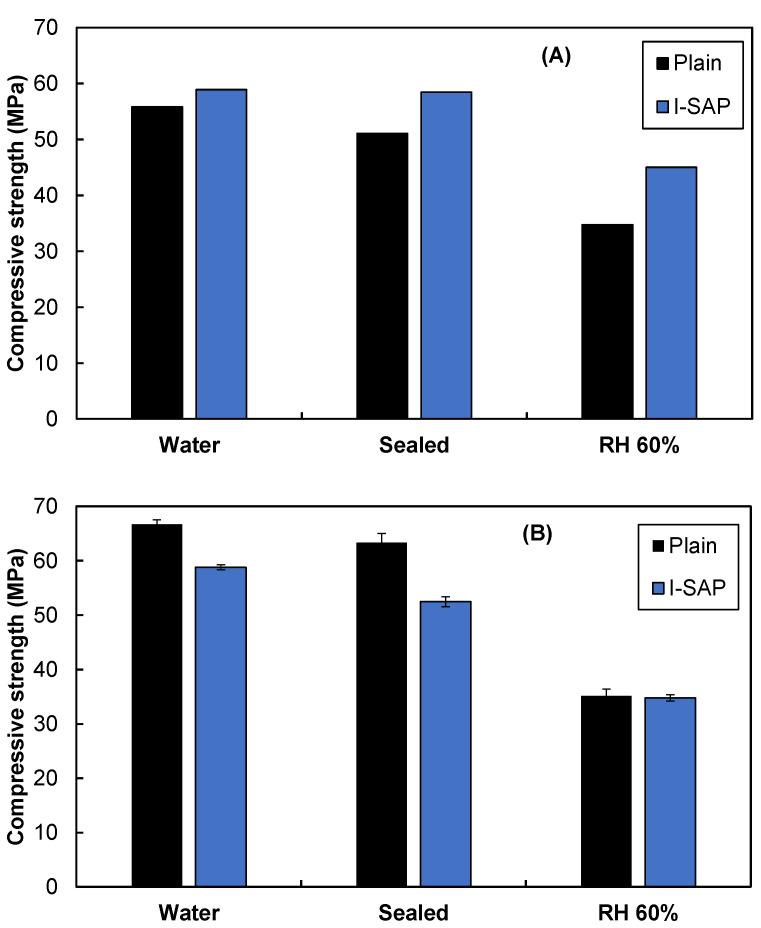
Compressive strength of (**A**) concrete, and (**B**) cement paste under different curing conditions.

As discussed in Section 3.1, the conventional SAP has lower flowability than plain or I-SAP, and thus, additional water is necessary to maintain the flowability, which may reduce the strength. Therefore, in this study, the compressive strength and microstructure of cement paste and concrete with I-SAP was compared with plain concrete and paste, and C-SAP was not considered as it lowers strength [8,11,28]. Figure 4 shows the compressive strength of concrete and hydrated cement paste under water, sealed, and at 60% RH curing conditions. The concrete with I-SAP showed a higher strength as compared to the plain one. The strength is seen to increase by 6%, 14%, and 30% under water, sealed, and RH 60% curing conditions, respectively, where 60% RH significantly impacts the strength development. On the contrary, the addition of SAP reduced the strength of the hydrated cement paste by 12%, 17%, and 1% in cement pastes cured under water, sealed, and RH 60% curing conditions, respectively. There was no significant difference in the strength of the paste cured at RH 60%. The results obtained here for hydrated Portland cement paste are consistent with those of previous studies [10,20,21,22,23,24,25,26,27,28,29]: SAP either reduces or does not alter compressive strength. The release of water from SAP forms voids, which decreases the hydrated cement strength [5,43]. However, a more detailed investigation of the microstructure is necessary to understand the strength gain in concrete, especially for concrete cured at an RH of 60%.

### 3.3. Effect of SAP on Microstructure

**Figure 5:**
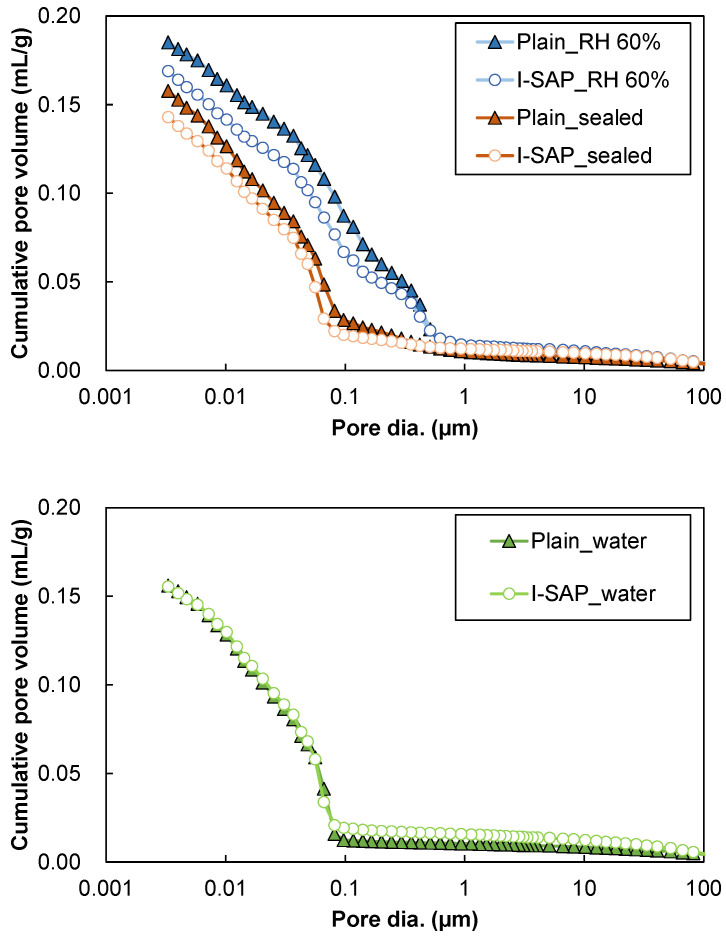
Cumulative pore volume of hydrated cement paste under different curing conditions.

**Figure 6:**
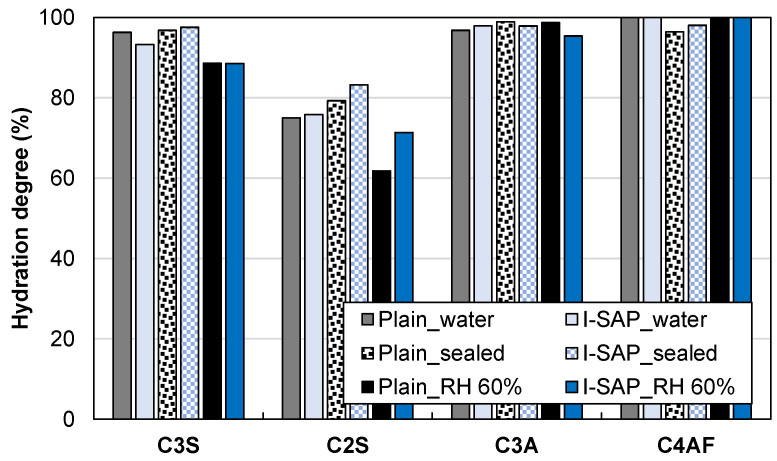
Effect of curing condition on hydration degree of individual clinker.

To evaluate the strength gain in concrete and loss in cement paste, the microstructures of the hydrated cement paste and concrete were analysed using various experimental techniques. A wide range (1–10 mm) of pore and void sizes exists in concrete, and thus, at least two methods (MIP and μCT) should be used to evaluate the pore structure in hydrated cement paste with SAP [48]. Figure 5 shows the cumulative pore volume of hydrated cement paste cured in water, sealed, and RH 60% conditions. The addition of SAP did not change the pore volume of cement paste cured in water, whereas it reduced the volume of pores diameter less than 600 nm in the seal-cured samples, which were cured in a 60% RH environment. In particular, the pore volume reduction was significant in this condition. This tendency is similar to that of the increase in the compressive strength of the concrete (Figure 4A). Therefore, the densification of the cement matrix, caused by hydration of clinker minerals, contributes to the strength development of concrete. The hydration rate of clinker minerals in the hydrated cement paste was calculated using XRD/Rietveld analysis, and the results are shown in Figure 6. The hydration degree of aluminate and ferrite is more than 95% with or without SAP in each curing condition, and thus, the effect of SAP on the reaction of these minerals is negligible. The hydration degree of alite in water and sealed curing is more than 93%, which is higher than that in the samples cured at RH 60%. Furthermore, SAP did not influence alite reaction. However, SAP increased the hydration degree of belite in the samples cured under sealed and RH 60% conditions by about 4% and 10%, respectively. The effect of SAP on belite hydration in the samples cured in water was insignificant as the curing provided enough water for hydration. Based on the reaction degree of the clinker minerals, the hydration of belite significantly contributes to the densification of the cement matrix, which reduces the porosity and increases the strength of concrete.

**Figure 7:**
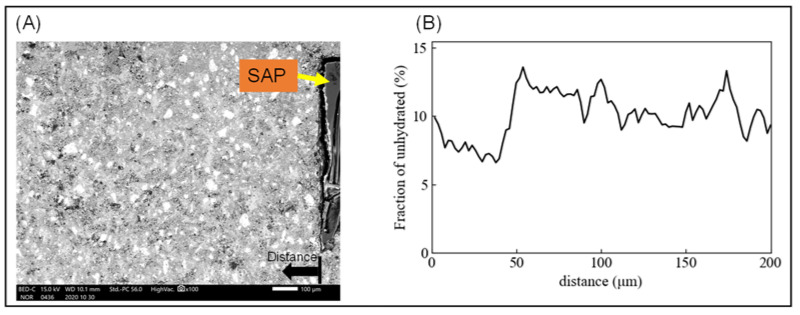
(**A**) SEM-BSE image, and (**B**) volume fraction of unhydrated cement from SAP surface.

The XRD/Rietveld results showed that the addition of SAP increased the hydration rate, especially belite, but it should be examined whether the hydration rate increased in the vicinity of SAP or throughout the entire cement matrix. Scanning electron microscopy (SEM) using BSE was conducted for the samples cured in the RH 60% environment, and a significant difference in belite hydration was seen. The results are shown in Figure 7. The volume fraction of unhydrated cement was determined by brightness separation [49], and the average of the three samples is shown in Figure 7B. The volume ratio of unhydrated cement is lower in the region 50 μm from the SAP surface than above it. In other words, it is considered that the hydration rate is high around the SAP surface. This is consistent with the results of Yanliang et al. [50], who reported that the volume fraction of high intensity unhydrated cement is low 50 μm from the SAP surface. It is considered that the addition of SAP supplies the absorbed water to a region of approximately 50 μm from the SAP surface, and as a result, the hydration reaction of cement mainly belite was enhanced, resulting in the cement matrix densification.

**Figure 8:**
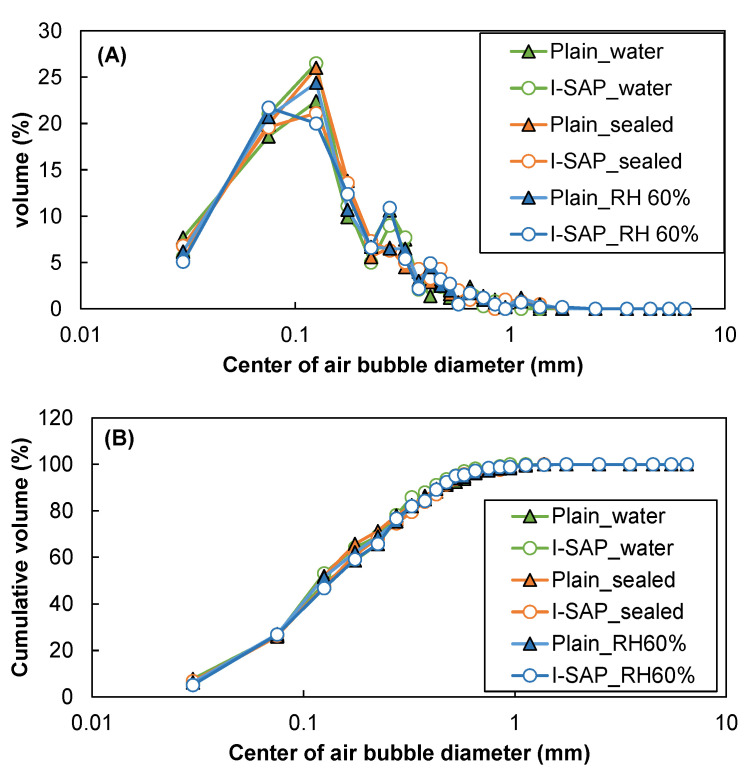
(**A**) distribution, and (**B**) cumulative volume of air bubbles in concrete.

The presence of air voids in concrete or large voids due to the release of water from SAP could influence the microstructure and eventually the compressive strength. A linear transverse method was used to measure the air voids in the concrete, and the results are shown in Figure 8. The addition of SAP did not affect the distribution of air voids or the total volume of air, and neither were the curing conditions. It has been reported that the air void distribution increases with the addition of SAP in mortar [51], which does not occur here due to the considerable delay in water absorption by SAP (Figure 2). It can be inferred that based on the results from MIP, XRD, SEM, and air voids, the densification of the cement matrix due to the hydration of cement around the SAP particles contributes to the strength enhancement in concrete.

**Figure 9:**
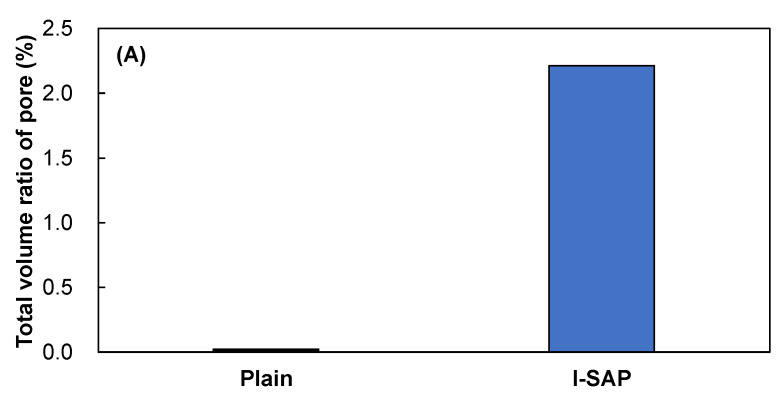

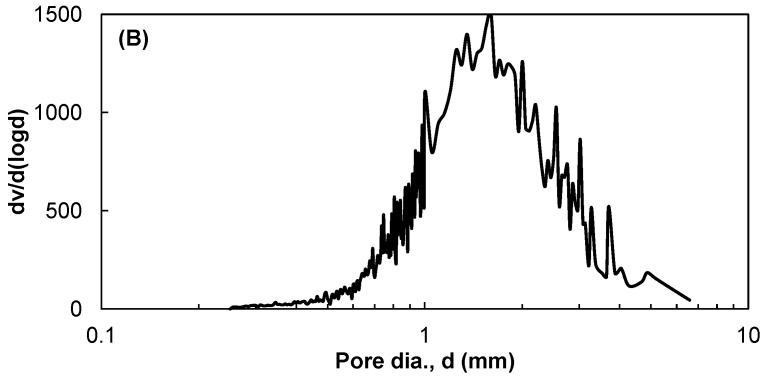
(**A**) total volume ratio of pore, and (**B**) pore size distribution.

The large pores or voids formed by the release of water from SAP could reduce the strength of hydrated cement paste. Because SAP voids were too large to be detected by MIP measurements [27,52], μCT was performed on the hydrated cement paste samples cured in sealed conditions. Thereafter, a 3D particle size analysis was conducted for the obtained μCT images, and the results are shown in Figure 9. The volume ratio of SAP pores is shown in Figure 9A, which is approximately 2.2%. A similar measurement was conducted for the samples without SAP, and the ratio was approximately 0.022%, which might be due to the air formed during the mixing of cement paste. It is shown that the pores of SAP voids are 100 times larger than those of SAP-free mixture. Furthermore, the void sizes are mostly 0.1–10 mm (Figure 9B). Therefore, in the hydrated cement, SAP addition decreases the pores on the nanoscale, owing to the reaction of unhydrated cement (Figure 5), but the voids created by SAP control the strength of the hydrated cement paste.

**Figure 10:**
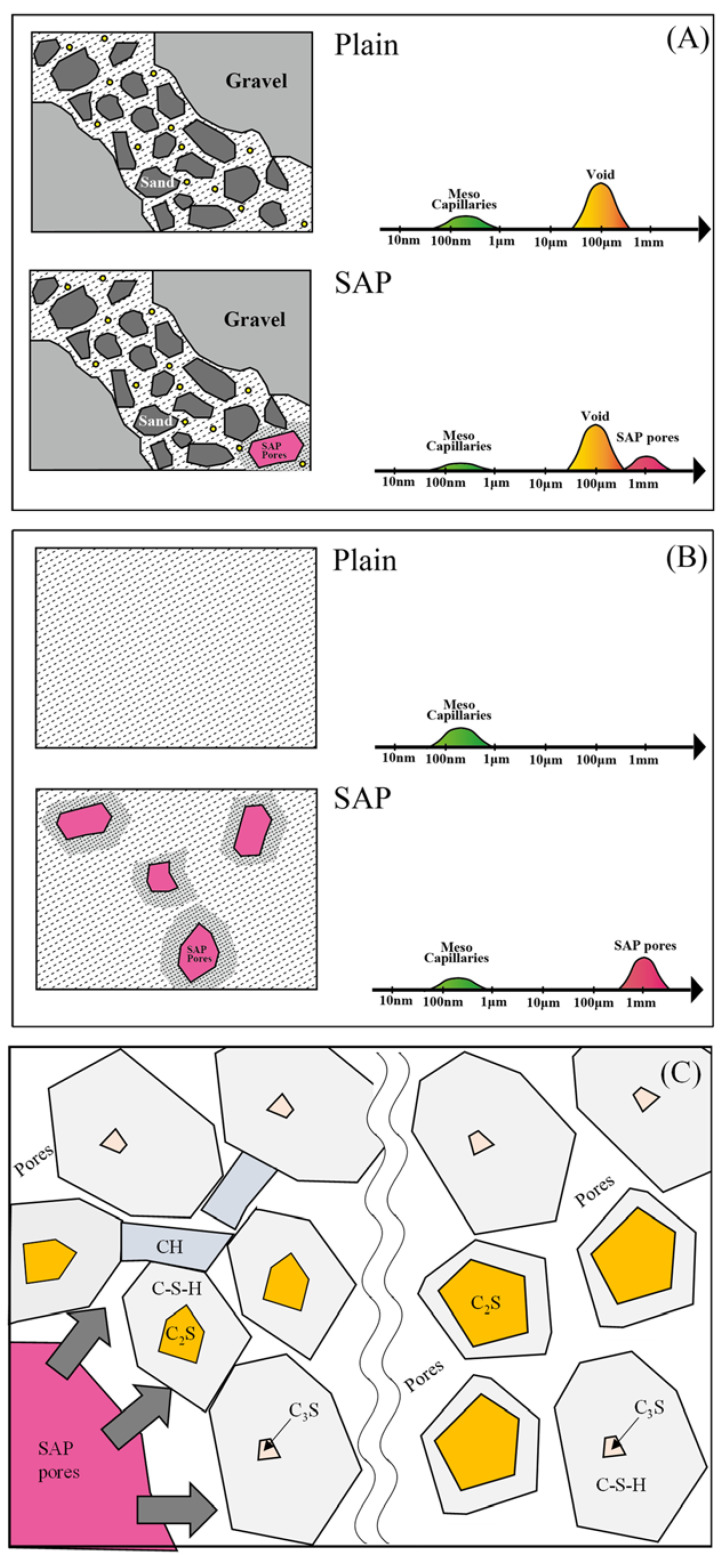
Schematic diagram of microstructure of (**A**) concrete; (**B**) cement paste with and without SAP; and (**C**) mechanism of cement matrix densification.

Figure 10 shows the schematic diagram of the microstructures of concrete and cement paste; the left represents the microstructure, whereas the pore size distribution is illustrated in the right. Moreover, the proposed mechanism for cement paste densification by the release of water from SAP is shown in Figure 10C. As seen from the material design and μCT data, concrete consists of voids three times larger than the pores generated by the SAP. If the air void does not change in the concrete, the large pores created by the SAP and its effect on strength can be ignored. Therefore, the strength gain in concrete with SAP cured in sealed and at RH 60% conditions is due to the cement matrix densification through the progress of hydration. The reason for the decrease in strength in the cement paste could be due to the pores formed by the water-released SAP despite the denser microstructure of the cement matrix. It is believed that in the cement paste cured at RH 60%, the SAP pores are offset by the hydration progress, resulting in the nearly equal compressive strength of samples with and without SAP (Figure 4B).

## 4. Conclusions

In this study, a delayed absorption type SAP (I-SAP) was fabricated, and its characteristics and effects on its compressive strength after 28 days were investigated. Moreover, the influence of the curing condition on strength was studied. The following conclusions can be drawn:

- I-SAP showed a higher absorption capacity in the pore solution than in deionised water and strongly influenced the compressive strength of concrete and hydrated cement paste.
- V-funnel time, indicating flowability, was 8.3 times higher for concrete with C-SAP than for plain concrete, whereas it was around 1.85 times higher for plain for concrete with I-SAP.
- The strength of the concrete with I-SAP increased compared to that without I-SAP under each curing condition, especially for the concrete cured under sealed conditions and 60% RH environment. An increase of 14% and 30% was seen with respect to plain concrete in sealed and at 60% RH curing conditions, respectively.
- The addition of I-SAP reduced the strength of hydrated cement paste cured in water and sealed conditions. The strength of the I-SAP-added paste in the samples cured at 60% RH was equivalent to that of the plain concrete.
- Similar air void distribution was seen regardless of the curing method or presence of I-SAP. The porosity of the cement paste decreased with the addition of SAP in the sealed and RH 60% curing conditions, which supports the strength development of concrete.
- The inclusion of SAP mainly promoted the hydration reaction of belite, suggesting that the water in SAP affects the reaction approximately 50 μm from the SAP surface. Therefore, the strength enhancement of concrete by adding SAP was due to the cement matrix densification.
- The developed I-SAP can be applicable in producing concrete without adding extra water to mitigate autogenous and drying shrinkage while maintaining or enhancing compressive strength.

## Figures and Tables

**Table 1:** Chemical composition of OPC.

Chemical Composition	Mass %
CaO	67.07
SiO_2_	20.38
Al_2_O_3_	5.12
Fe_2_O_3_	3.05
SO3	1.86
MgO	1.30
K_2_O	0.36
Na_2_O	0.30
TiO_2_	0.29
P_2_O_5_	0.20
MnO	0.05

**Table 2:** Proportions, constituents, and properties of concrete mixtures.

	Reference	SAP-Concrete
Coarse aggregate (kg/m^3^)	930	930
Fine aggregate (kg/m^3^)	830	830
Cement (kg/m^3^)	380	380
Water (kg/m^3^)	170	170
Superplasticizer (% BWOC)	0.95	1.10
SAP (kg/m^3^)	0	1.14
w/c	0.45	0.45
Slump (cm)	22.0	23.0
Slump flow (cm)	408	428
Air (%)	4.5	4.5
Setting time: Initial (h)	6:21	6:40
Final (h)	8:09	8:32

## Data Availability

Not applicable.
